# Antisclerosin in Arteriosclerosis

**Published:** 1904-02

**Authors:** S. Goldschmidt

**Affiliations:** Physician Bad Reichenhall


### ABSTRACTS.

### Antisclerosin in Arteriosclerosis.

By DR. S. GOLDSCHMIDT,
Physician Bad Reichenhall.

[*Deutsche Praxis Zeitschrift für praktische Aerzte*, No. 19, 1903.]

During the past few months I have had occasion to employ a remedy for which very good results in arteriosclerosis are claimed and which is called antisclerosin. It is composed of various blood salts and is manufactured by W. Natterer, of Munich, who placed a quantity of it at my disposal. I have tried it altogether in 13 cases. I selected patients with advanced arteriosclerosis, both central,—sclerosis of the heart,—and peripheral. The results show that the remedy is excellent, for its effects were not only symptomatic and temporary, but sometimes also permanent.

Case I.—A man of 65, with marked sclerosis of heart and arteries. He complained of beating of the temples, muscæ volitantes, occasional tinnitus, palpitation, and the like. The action of antisclerosin was very surprising. After taking two tablets three times daily for 10 days all the troublesome phenomena disappeared. The pulse showed a diminution in hardness.

Case II.—Aged 50. Very advanced sclerosis and arhythmia of the heart; occasional pseudoasthmatic attacks. Antisclerosin, same dose. Returned home after 14 days. Condition has remained satisfactory.

Case III.—Patient confined to bed. Pulse extraordinarily hard and slow, 60 to the minute. Heart enlarged in all dimensions; tone on auscultation, loud and ragged. Loud murmurs at all the orifices and edema of feet. Diuresis small; no albumin. Moderate emphysema with a little catarrh. Digitalis. Eight days later, in somewhat better condition; antisclerosin. One week later edema of the feet had disappeared, dyspnea had gone and patient got up. Now feels very well under antisclerosin and $CO_2$ baths. Cardiac murmurs remain. Heart configuration unchanged.

Case IV.—Widow of 68. Central and peripheral sclerosis, cardiac palpitation and rheumatoid pains. Antisclerosin for 10 days. Marked improvement in palpitation. Objectively no change was noticeable.

Case V.—Countess M., 61. Suffered for many years from arhythmia and far advanced sclerosis of the heart and arteries; edema of the lower extremities. Stenocardiac attacks. September 10, antisclerosin. On the 15th she had a fainting spell lasting for several minutes. When she regained consciousness amnesic aphasia had appeared. The question: had the apoplexy any connection with the antisclerosin? I can positively answer in the negative. On the 18th the patient indicated pains in the head and the heart. Antisclerosin, which had been stopped for 3 days, resumed. Two days later the heart became quieter. Arhythmia was noticeably less, the cardiac contractions were much more regular and the pains had disappeared. Ice bag and the Leiter apparatus had been used in addition to antisclerosin. No more complaints of stenocardia. The cerebral trouble is slowly improving.

The other eight cases present a similar history and need not be reported.

In all cases the dose was 2 tablets thrice daily, an hour before meals or with meals. The former method should be used if the latter does not give results. Diet consisted mostly of vegetables with little or no alcohol. All kinds of baths were given.

The favorable effects were doubtless due to antisclerosin. In some few cases the iodine preparations might have done as well, but would have been slower in action. Iodine has, besides, unpleasant by-effects which prevent its use in very many cases. There were no such idiosyncratic phenomena with antisclerosin.

